# Polyamines: Bio-Molecules with Diverse Functions in Plant and Human Health and Disease

**DOI:** 10.3389/fchem.2018.00010

**Published:** 2018-02-05

**Authors:** Avtar K. Handa, Tahira Fatima, Autar K. Mattoo

**Affiliations:** ^1^Department of Horticulture and Landscape Architecture, Purdue University, West Lafayette, IN, United States; ^2^Sustainable Agricultural Systems Laboratory, Henry A. Wallace Beltsville Agricultural Research Center, Agricultural Research Service (ARS-USDA), Beltsville, MD, United States

**Keywords:** biogenic amines, nutrition, metabolism, polyamines and human diseases, vegetables, fruits, cereals, meats

## Abstract

Biogenic amines—polyamines (PAs), particularly putrescine, spermidine and spermine are ubiquitous in all living cells. Their indispensable roles in many biochemical and physiological processes are becoming commonly known, including promoters of plant life and differential roles in human health and disease. PAs positively impact cellular functions in plants—exemplified by increasing longevity, reviving physiological memory, enhancing carbon and nitrogen resource allocation/signaling, as well as in plant development and responses to extreme environments. Thus, one or more PAs are commonly found in genomic and metabolomics studies using plants, particulary during different abiotic stresses. In humans, a general decline in PA levels with aging occurs parallel with some human health disorders. Also, high PA dose is detrimental to patients suffering from cancer, aging, innate immunity and cognitive impairment during Alzheimer and Parkinson diseases. A dichotomy exists in that while PAs may increase longevity and reduce some age-associated cardiovascular diseases, in disease conditions involving higher cellular proliferation, their intake has negative consequences. Thus, it is essential that PA levels be rigorously quantified in edible plant sources as well as in dietary meats. Such a database can be a guide for medical experts in order to recommend which foods/meats a patient may consume and which ones to avoid. Accordingly, designing both high and low polyamine diets for human consumption are in vogue, particularly in medical conditions where PA intake may be detrimental, for instance, cancer patients. In this review, literature data has been collated for the levels of the three main PAs, putrescine, spermidine and spermine, in different edible sources—vegetables, fruits, cereals, nuts, meat, sea food, cheese, milk, and eggs. Based on our analysis of vast literature, the effects of PAs in human/animal health fall into two broad, Yang and Yin, categories: beneficial for the physiological processes in healthy cells and detrimental under pathological conditions.

## Introduction

Polyamines (PAs) are aliphatic polycations ubiquitously present in all tissues and all cell types examined in animals and plants. PAs have specific and diverse roles in multiple cellular processes, including apoptosis, cell division and differentiation, cell proliferation, DNA and protein synthesis, gene expression, homeostasis, signal transduction (Cohen, 1998; Kaur-Sawhney et al., 2003; Kusano et al., 2008; Anwar et al., 2015; Pegg, 2016). Although, interest in the biology and mechanism of action of PAs was sporadic since the initial discovery of spermine (Spm) phosphate crystals in human semen (~1678 AD), it is now known that PAs and their analogs have functional implications in plant life as well as in various aspects of human/animal health and disease. For instance, their role(s) are recognized in gastroenterology (Pfeffer et al., 2001), programmed cell death (PCD) (Pfeffer et al., 2001; Pignatti et al., 2004; Moschou and Roubelakis-Angelakis, 2014; Cai et al., 2015); parasitology (Bacchi and Yarlett, 2002), cerebral stroke and other disorders (Tomitori et al., 2005), oxidative stress (Tomitori et al., 2005; Agostinelli et al., 2006; Cona et al., 2006), oncology (Casero and Marton, 2007), and obesity (Jell et al., 2007). Likewise, in plants PAs feature in a large range of processes including plant growth and development and, in particular, stress/senescence responses (Cohen, 1998; Kaur-Sawhney et al., 2003; Capell et al., 2004; Tang and Newton, 2005; Handa and Mattoo, 2010; Bitrián et al., 2012; Pathak et al., 2014; Tiburcio et al., 2014; Anwar et al., 2015; Liu et al., 2015; Gupta et al., 2016; Sobieszczuk-Nowicka, 2017). These findings have intensified research on elucidating function(s) of PAs in pharmacology and medicine (Bachrach and Wang, 2002; Janne et al., 2004; Agostinelli et al., 2006; Guerra and Rubin, 2016; Pegg, 2016), modulating pre- and postharvest biology (Paschalidis and Roubelakis-Angelakis, 2005; Uemura et al., 2005; Nambeesan et al., 2010, 2012) and abiotic and biotic stresses in plants (Yamaguchi et al., 2006; Prabhavathi and Rajam, 2007; He et al., 2008; Wen et al., 2008; Alcazar et al., 2010a,b; Mattoo et al., 2015).

Main PAs in mammalian cells and plants are putrescine (Put), spermidine (Spd) and spermine (Spm), with thermo-Spm (T-Spm) discovered in plants to be important against abiotic stress. Other than these PAs, flowering plants also synthesize cadaverine, 1,3-diaminopropane, and other modified forms. Other amines include cadaverine, homoSpd, norSpd, homoSpm, norSpm, thermoSpm, aminopropyl homoSpd, and methyl Spd, which are less predominant (Grimes et al., 1986; Tiburcio et al., 1993; Hamana et al., 1998; Martin-Tanguy, 2001). The presence of branched and long PAs generally present in legumes and graminae seeds are thought to protect the seed from water deficit, while di-, tri-, and tetra-PAs in aquatic plants may regulate osmotic stress during submergence in water (Hamana et al., 1998).

Biochemical roles of PAs have been recognized in several processes, including the synthesis, maintenance of structure, stability and functions of proteins and nucleic acids (Park, 2006). Examples include, but are not limited to, the posttranslational maturation of Eukaryotic Translation Initiation Factor 5A (eIF5A) by the conversion of Lys to Spd-derived hypusine, which is essential for translation of mRNAs of several proteins containing polyproline tracts or triplets of PPX (X being Gly, Trp, Asp, or Asn). These proteins are essential for viability playing roles in DNA binding, transcription, RNA splicing and turnover, cell signaling, actin/cytoskeleton-associated functions, nucleocytoplasmic transport and apoptosis (Nishimura et al., 2012; Caraglia et al., 2013; Mandal et al., 2014; Sievert et al., 2014; Mathews and Hershey, 2015; Pällmann et al., 2015). PA binding to the inwardly rectifying K^+^ (Kir) channels facilitates movement of K^+^ into the cell and affects resting membrane potential, electrolyte balance, and cardiac and electrical activity and controlling the electrical excitation in many types of cells (Hibino et al., 2010; Baronas and Kurata, 2014). Spd binding to NMDAr, AMPAr and kainite influence their function including in animal/human memory (Williams et al., 1989; Bowie and Mayer, 1995; Williams, 1997). PAs also regulate C, transient receptor potential cation (TRPC) channels and connexins to regulate the gastrointestinal smooth muscle excitability and contractility (Pegg, 2016). Genomic and metabolomic studies have identified gene medleys and metabolic pathways regulated by PAs, impacting cellular metabolism and involving processes in subcellular compartments from nucleus to mitochondria and cytoplasm. In plants as well, PAs abound in cell organelles, including nucleus, chloroplast, mitochondria and chromoplasts in different forms—as free amines, conjugated to small molecules, and bound to larger macromolecules (Martin-Tanguy, 2001).

Metabolite profiling has identified Spd and Spm as regulators of nitrogen (N) and carbon (C) metabolism, causing accumulation of other N forms such as Glu, Gln and Asn. The sensing of PAs and their signaling responses are common responses of plant roots, leaves and trees to exogenous N (Rennenberg et al., 1998; Foyer and Noctor, 2002; Bauer et al., 2004; Mattoo et al., 2006; Mattoo and Handa, 2008). The N assimilation and C metabolism in different plant organs seems conserved, contributing to the totipotent nature of plant cells. Regulation of N and C use pathways also became prominent through studies with transgenic poplar cell culture lines that accumulated high Put levels in comparison to their low Put-producing line. Higher Put levels were associated with increased flux of glutamate into ornithine, while this also caused concurrent enhancement in glutamate production involving more N and C assimilation (Page et al., 2016).

Also, upon reaching a threshold in cellular PAs, cells down-regulate assimilation of nitrate-derived N (Provan et al., 2000; Shen and Huber, 2006), supporting the notion that PAs may also be signals of nitrogen-replete conditions. One way of achieving this regulation is by stimulating specific protein–protein interactions (Aitken, 1996; Provan et al., 2000; Bridges and Moorhead, 2004; Shen and Huber, 2006; Garufi et al., 2007). A positive role of Put in mitigating a destructive disease in the form of mango malformation further suggests that PAs regulate specific developmental processes in plants (Singh et al., 2014). Growth and life span in eukaryotes is regulated by a major nutrition and energy sensor called Target of Rapamycin (TOR). Among many functions of TOR include regulation of anabolic pathways involving polysome integrity, translation of mRNA and ribosome biogenesis (Proud, 2004; Tavernarakis, 2008; Ren et al., 2012), functions similar to PAs. It is interesting that PA biosynthesis in plants seems connected to TOR function in the plant model Arabidopsis (Ren et al., 2012). In this context, PAs have been implicated in TOR signaling and prostate cancer (Zabala-Letona et al., 2017). Together, these findings demonstrate that some functions of PAs are conserved, providing parallels between plants and humans.

## Biosynthesis of PAs

**Figure 1:**
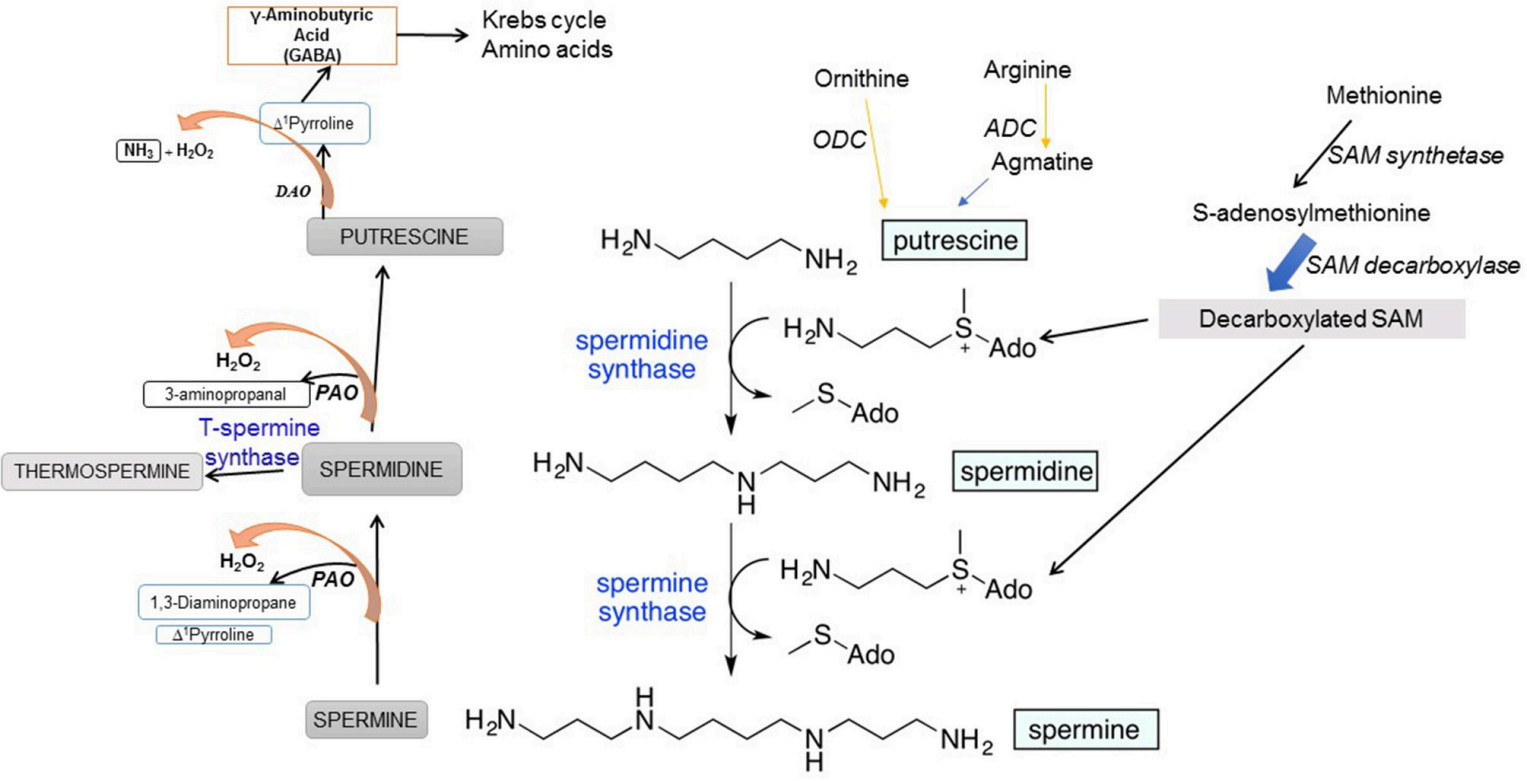
Polyamine biosynthetic and the back-conversion pathways in plants.

PA biosynthesis initiates from two amino acids, arginine (Arg) and ornithine (Orn). In animals, Arg is first converted by mitochondrial arginase to Orn, which is then decarboxylated by ODC to synthesize Put. Plants use an additional pathway involving arginine (Arg) decarboxylation by Arg decarboxylase (ADC) to produce Put (Fig. 1). Decarboxylated arginine—agmatine—is synthesized by plants and by many bacteria, including the intestinal flora but not in mammals (Coleman et al., 2004; Pegg, 2016) where its source is either food ingested or the intestinal microflora. Decarboxylation of S-adenosylmethionine (SAM), catalyzed by SAM decarboxylase (SAMDC), yields aminopropyl group that acts as a substrate together with Put for Spd synthase and generates Spd followed by the incorporation of another aminopropyl group (also from decarboxylated SAM) catalyzed by Spm synthase to form Spm (Figure 1). Spd is also converted to a Spm isomer, thermospermine (T-Spm), a reaction catalyzed by T-Spm synthase (Figure 1). T-Spm has been shown to be required for normal growth and development, promoting stem elongation and suppresses auxin-induced xylem differentiation in *Arabidopsis* (Takano et al., 2012; Yoshimoto et al., 2016 and references therein). ODC in mammals requires pyridoxal phosphate (PLP) as a cofactor (Lee et al., 2007) while other SAMDCs have covalently bound pyruvate for catalysis (Tolbert et al., 2001). In addition to the difference between plants and animals in the initial reactions catalyzing the formation of Put, the latter also synthesize ODC antizyme that inhibits ODC enzymatic activity and regulates the Put homeostasis. The antizyme inhibits ODC and also targets the latter to the 26S proteasome for degradation—a process in animal cells to maintain cellular homeostasis of ODC activity (Wallace et al., 2003; Janne et al., 2004; Igarashi and Kashiwagi, 2010; Perez-Leal and Merali, 2011).

Polyamine biosynthetic pathway and genes encoding the enzymes involved have been identified and characterized in both prokaryotes and eukaryotes (Coleman et al., 2004; Kusano et al., 2007; Pegg, 2016). In *Lathyrus sativus*, an additional/alternate pathway of Spd synthesis exists which involves formation of a Schiff base between aspartic 4-semialdehyde with Put to yield carboxy-Spd as an intermediate that undergoes a pyridoxal phosphate-dependent enzymatic decarboxylation to give rise to Spd (Srivenugopal and Adiga, 1980). Such a pathway is known to be present in prokaryotes and was demonstrated and characterized in *Vibrio cholera* (Lee et al., 2009).

## Comparison of the content of PAs in plants and animals

The consumer demand for bountiful nutritious produce has intensified since the recognition that biomolecules such as antioxidants (including phenolic compounds), vision-improving carotenoid β-carotene, and anti-cancerous carotenoid lycopene have the potential to enhance quality of life in humans and animals. Thus, due to their anti-oxidant and anti-inflammatory attributes, PAs as well have been implicated in beneficial effects on human health (Lagishetty and Naik, 2008). However, PA levels generally decline with aging in most organisms, from bacteria to mammals, which may play a significant role in aging and associated diseases. The aging-associated decline in PA levels is reversed by dietary PA supplementation of a healthy population (Soda et al., 2009b). Studies on implications of food-based PAs for animal growth and health found them to be essential for normal metabolism in rat tissues and organs that had been stimulated to grow by metabolic signaling (Bardócz et al., 1993). Biological implication was studied using human population, which proposed that both endogenous and dietary PAs could be useful for post-operation patients during wound healing, and for the growth and development of neonate's digestive system (Kalac and Krausova, 2005).

Fruits and their juices, sauerkraut, ketchup, frozen green peas and fermented soybean products contain high levels, above 40 mg kg^−1^, while legumes (mainly soybean), pear, cauliflower and broccoli commonly accumulate high Spd content than Spm, usually above 30 mg kg^−1^ FW (Kalac and Krausova, 2005). Based on the published literature, low levels of Put are typical for well-treated foods of animal origin in contrast to foods of plant origin (Kalac and Krausova, 2005). Tables 1, 2 summarize the distribution of different PAs in selected plants and animals found in the literature. The reported quantities are given as mg.kg^−1^ in their descending order (Nishibori et al., 2007; Ali et al., 2011).

**Table 1:** Polyamines content in vegetables, fruits, grains, and other plant based diet.

**PUT (mg/Kg)**			**SPD (mg/Kg)**			SPM^*^ (mg/Kg)		
**Food item**	**Mean**	**Range**	**Food item**	**Mean**	**Range**	**Food item**	**Mean**	**Range**
**VEGETABLES**								
Green pepper	54.7^a^	31.2–84	Soybean, dried	207.0^e^	–	Soybean, dried	69.0^e^	–
Soybean, dried	41.0^e^	–	Soybean, dried	128.0^g^	–	Red kidney beans	24.3^b^	22.8–25.7
Green peas	32.4^a^	5.6–51.2	Mushroom	88.6^a^	62.4–139.3	Pumpkin	18.4^a^	6.6–41.4
Aubergine, eggplant	31.7^c^	–	Shiitake mushroom	56.4^a^	33.2–71.8	Broccoli, fresh	9.9^f^	5.8–15.9
Green onion	24.6^d^	–	Green peas	49.5^a^	4.5–94.5	Green pepper	9.0^a^	4–20.8
Okra	21.7^d^	–	Green soybeans	48.5^a^	34.8–67.2	Broccoli	8.7^a^	5.6–12.5
Lettuce	20.7^a^	10.2–42.4	Lettuce	44.0^a^	14.8–104.1	Burdock root	8.7^a^	3.0–13.7
Bean sprout	18.2^a^	15.3–24.6	Broccoli, fresh	41.3^f^	24.5–51.8	Japanese hornwort	6.7^a^	2.0–15.9
Potato, Fresh	17.6^e^	–	Enoki mushroom	32.5^a^	24.9–38.6	Green peas	6.5^a^	4.2–8.7
Eggplant	17.5^a^	11.2–22.0	Cauliflower, fresh	31.2^g^	17.1–42.8	Rucola (Arugula)	6.2^d^	–
Celery	17.1^a^	8.5–23.1	Lotus root	30.5^a^	17.2–51.4	Cauliflower, fresh	6.1^f^	4.6–8.9
Soybean, dried	17.0^g^	–	Chickpea	28.8^c^	–	Kidney French bean	5.9^a^	2.2–10.9
Japanese hornwort	15.7^a^	5.5–37.1	Cauliflower, fresh	28.3^g^	21.3–39.3	Garlic	5.8^a^	3.6–7.1
Carrot	14.8^a^	8.0–24.7	Celeriac (Knob Celery)	26.7^g^	19.7–34.7	Asparagus	5.7^a^	0.2–12.9
Cucumber	13.1^a^	2.1–25.8	Cauliflower, fresh	24.8^b^	21.7–27.8	Pea (Field)	5.7^a^	2.4–8.4
Green soybeans	13.0^a^	5.9–19.2	Broccoli	22.7^a^	16.1–28.3	Komatsuna	5.1^a^	2.4–8.2
Tomato	10.6^e^	–	Shimeji mushroom	22.2^a^	10.8–37.0	Green soybeans	4.0^a^	2.2–6.8
Burdock root	9.8^a^	1.3–25.2	Yam	20.9^a^	3.3–43.5	Celery	3.8^a^	1.6–5.4
Potato, Fresh	9.7^b^	9.5–9.9	Welsh onion	20.5^a^	9.4–27.4	Spinach	3.8^a^	3.0–5.4
Potato, Fresh	9.7^f^	5.8–12.8	Red kidney beans	19.5^b^	19–20	Mushroom	3.4^a^	3–4.5
Shimeji mushroom	9.5^a^	5.3–13.3	Okra	18.6^d^	–	Lettuce	3.2^a^	0.4–12.9
Broccoli, fresh	9.0^f^	7–10.5	Spinach	17.9^a^	14.0–22.3	Potato, Fresh	3.0^b^	2.8–3.2
Cucumber	8.7^e^	–	Asparagus	17.4^a^	2.1–32.2	Potato	2.8^a^	0.6–4.6
Parsley	8.7^a^	4.0–13.0	Komatsuna	17.1^a^	1.6–29.3	Potato, Fresh	2.6^f^	0.8–4.0
Potato	7.2^b^	5.8–10.4	Green onion	17.0^d^	–	Cabbage	2.4^a^	0.8–6.6
Cucumber	6.9^g^	5.5–8.7	Pea (Field)	16.7^a^	11.6–26.1	Cauliflower, fresh	2.4^b^	2–2.8
Pumpkin	6.6^a^	3.2–10.8	Burdock root	16.6^a^	2.6–35.5	Carrot	2.4^f^	2–2.8
Broccoli, fresh	6.4^f^	3.4–10.8	Japanese hornwort	16.0^a^	3.6–40.8	Welsh onion	1.8^a^	1.0–2.8
Onion	6.4^b^	5.5–7.2	Cucumber	15.5^e^	4.5–32.5	Parsley	1.8^a^	1.4–2.2
Celeriac (Knob Celery)	6.1^g^	3.7–7.7	Celery	14.2^a^	11.3–18.8	Red pepper	1.7^c^	–
Lettuce	5.6^f^	4.5–7.3	Potato, Fresh	13.5^e^	–	Carrot	1.4^a^	1.2–1.8
Asparagus	5.4^a^	3.2–6.6	Kidney French bean	11.8	4.1–27.4	Taro	1.2^a^	0.4–1.6
Cauliflower, fresh	5.3^f^	3.3–8.9	Green pepper	11.6^a^	7.7–17.8	Cucumber	1.2^e^	0–2.8
Cauliflower, fresh	4.9^g^	2.2–7.6	Potato, Fresh	11.3^f^	8.3–13.6	Chickpea	1.2^c^	–
Enoki mushroom	4.4^a^	2.2–6.6	Potato, Fresh	11.2^b^	11–11.3	Onion	1.0^b^	0.8–1.2
**FRUITS**								
Orange	137.0^f^	119–153	Mango	30.0^d^	–	Pear	–^b^	8.1–49.3
Orange	117.6^b^	95.1–140	Melon	11.7^d^	–	Peach	5.1^d^	–
Orange	117.0^e^	–	Banana	11.3^h^	–	Avocado	4.0^d^	–
Mandarin	122.0^f^	67.3–200	Avocado	10.2^d^	–	Mango	3.2^d^	–
Mango	80.0^d^	–	Orange	8.4^b^	8.8–9.7	Pineapple	2.2^c^	–
Lime	41.0^d^	–	Peach	6.1^d^	–	Lime	1.8^d^	–
Chinese citron	27.8^a^	20.6–34.7	Banana	5.8^a^	4.2–8.2	Orange	1.6^e^	–
Pear	24.0^b^	23.6–24.2	Kiwi	5.4^c^	–	Banana	1.5^h^	–
Orange	21.5^a^	6.4–36.4	Papaya	5.3^d^	–	Kiwi	1.5^c^	–
Banana	15.3^h^	–	Fig	5.2^d^	–	Cherry	0.8^a^	0.4–2.0
Banana	12.3^a^	11.2–13.8	Lime	5.0^d^	–	Banana	0.6^a^	0–1.6
Papaya	4.6^d^	–	Orange	4.1^e^	0.4–11.6	Mandarin	0.4^f^	0–3.0
Dates (dry)	2.8^c^	–	Pineapple	4.0^c^	–	Strawberry	0.4^a^	0.2–0.6
Fig	2.2^d^	–	Pear	3.0^c^	–	Apple	0.2^a^	0–0.4
Cherry	1.6^a^	0.4–4.3	Peach	2.7^c^	–	Dates (dry)	0.2^c^	–
Kiwi	1.2^c^	–	Apple	2.5^b^	2.2–2.8	Orange	0.2^f^	0–1.4
Prune	1.1^d^	–	Mandarin	2.3^a^	0–0.5			
Strawberry	1.0^a^	0.8–1.2	Strawberry	2.0^a^	1.6–3.0			
**GRAINS**								
Maize fresh	50.7^a^	18.3–85.4	Corn	43.2^d^	–	Rice Brown	10.0^a^	–
Corn	–^d^	17.4–74	Maize grain	21.0^a^	8.0-39	Millet	9.7^d^	–
Rice Brown	5.0^a^	–	Whole grain	24.4^b^	21.3-27.4	Whole grain	8.1^b^	7.1–9.1
Whole grain	3.5^c^	–	Whole grain	15.8^c^	–	Whole grain	6.3^f^	3.4–8.7
Whole grain	3.4^f^	2.5–4	Whole grain	13.1^f^	10.2–14.8	Whole grain	4.5^c^	–
Millet	1.7^d^	–	Millet	9.1^d^	–	Rice, polished	4.1^e^	–
Rice, polished	0.9^e^	–	Rice Brown	6.4^a^	–	Maize grain	1.6^a^	0.2–5.1
Whole grain	0.7^b^	0.5–0.9	Rice, polished	3.9^e^	–	Corn	1.2^d^	–
Rice	0.2^a^	0.2–0.03	Rice	0.4^a^	0.3–0.6	Rice	0.6^a^	0.2–0.8
**OTHERS**								
Pistachio	18.2^a^	7.2–27.6	Tea, black leaves	38.1^b^	36.5–39.7	Tea, black leaves	59.0^b^	57.8–60.0
Tea, black leaves	15.3^b^	14.4–16.1	Dill	29.2^d^	–	Cashew	24.0^a^	18.4–28.3
Dill	12.7^d^	–	Hazelnut (dried)	21.0^c^	–	Black leaves	19.0^e^	–
Coffee, green	10.3^i^	9.1–16.3	Peanut	16.0^a^	12.7–19.1	Peanut	17.8^a^	13.3–25.7
Black leaves	7.0^e^	–	Peppermint	13.2^d^	–	Almond	13.5^a^	11.7–15.5
Peppermint	6.8^d^	–	Black leaves	11.4^e^	–	Pistachio	13.3^a^	–
Hazelnut (dried)	4.2^c^	–	Pistachio	11.0^a^	–	Dill	8.7^d^	–
Almond	1.6^a^	1.1–3.0	Almond	6.0^a^	5.1–7.4	Hazelnut (dried)	6.5^c^	–
Peanut	1.5^a^	0.8–2.8	Coffee, green	6.0^i^	5.1–6.8	Coffee, green	4.4^i^	4.2–7.3
Cashew	0.8^a^	0.3–1.2	Cashew	4.6^a^	3.2–5.2	Peppermint	2.0^d^	–

* *Spermine (Spm) values given do not discriminate between Spm and its isomer thermospermine (T-Spm) and therefore represent a total of Spm+T-Spm. Spm and T-Spm are not distinguishable in a standard PA analysis methods*.

a *Nishibori et al. (2007)*,

b *Bardócz et al. (1993)*,

c *Cipolla et al. (2007)*,

d *Nishimura et al. (2006)*,

e *Okamoto et al. (1997)*,

f *Eliassen et al. (2002)*,

g *Ziegler et al. (1994)*,

h *Lavizzari et al. (2006)*,

i *(Cirilo et al., 2003). – Data not detected/data not available*.

**Table 2:** Polyamines content in meat, seafood, dairy, and other animal based diet.

**PUT (mg/Kg)**			**SPD (mg/Kg)**			SPM^*^ (mg/Kg)		
**Food item**	**Mean**	**Range**	**Food item**	**Mean**	**Range**	**Food item**	**Mean**	**Range**
**MEAT**								
Fallow deer	38.0^h^	–	Pheasant	21^h^	–	Beef liver	197^d^	–
Roe deer	19.3^h^	–	Beef Raw, lean	19^b^	18.3–19.7	Pheasant	83^h^	–
Beef Raw, lean	10.1^f^	0.8–38.5	Red deer	17^h^	–	Duck	65.3^d^	–
Red deer	9.0^h^	–	Roe deer	14.7^h^	–	Raw chicken	62.6^e^	–
Beef Raw, lean	5.7^b^	5.5–5.9	Fallow deer	14.7^h^	–	Fallow deer	60.7^h^	–
Raw, lean	3.0^b^	2.9–3.1	Chicken thigh, raw	10.2^i^	–	Red deer	59.3^h^	–
Raw chicken	2.9^b^	2.8–2.9	Raw chicken	9.3^b^	9.1–9.4	Raw chicken	59.2^b^	58.8–59.9
Sirloin, raw	2.1^f^	0.6–12.8	Duck	8.4^d^	–	Roe deer	54.3^h^	–
Duck	1.7^d^	–	Chicken breast, raw	7.7^j^	–	Lamb	47.1^d^	–
Turkey wing	1.2^c^	–	Chicken thigh, raw	7.2^j^	–	Chicken	45.7^a^	37.8–54.6
Pork raw, lean	1.1^e^	–	Beef liver	6.8^d^	–	Beef Raw, lean	39.8^k^	28.7–44.6
Lamb	1.0^d^	–	Chicken	6.5^a^	4.5–8.2	Chicken thigh, raw	38^i^	–
Beef liver	1.0^d^	–	Beef Raw, lean	5.5^f^	2.6–12	Chicken breast, raw	36.8^i^	–
Beef	0.9^a^	0.4–1.3	Lamb	5.0^d^	–	Beef Raw, lean	36.4^b^	30.7–42.0
Chicken	0.7^a^	0.4–1.0	Chicken breast, raw	4.8^i^	–	Beef	31.1^a^	26.7–34.3
Ham	0.5^a^	0.2–2.0	Pork raw, lean	4.6^e^	–	Sirloin, raw	30.0^l^	–
Beef Raw, lean	0.5^e^	–	Pork raw, lean	3.9^b^	2.9–4.9	Beef Raw, lean	28.3^e^	–
Raw chicken	0.4^e^	–	Beef Raw, lean	3.1^k^	1.9–4.2	Beef Raw, lean	27.3^f^	16.8–26
Pork	0.3^a^	0.1–0.5	Raw chicken	2.9^e^	–	Pork	26.1^a^	19.4–28.9
Chops, raw	0.2^f^	0–0.7	Pork Chops, raw	2.8^f^	2.0–4.1	Chops, raw	22.4^f^	14.5–34.5
			Beef Raw, lean	2.6^e^	–	Chicken breast, raw	17.2^j^	–
			Beef	2.3^a^	1.1–4.0	Sirloin, raw	17^f^	6.1–29.1
			Sirloin, raw	2.2^f^	1.0–3.0	Chicken thigh, raw	16.2^j^	–
			Sirloin, raw	1.5^l^	–	Turkey wing	13.8^c^	–
			Ham	1.4^a^	0.7–3.7	Ham	10.1^a^	2.0–31.3
			Turkey wing	1.4^c^	–			
**SEAFOOD**								
Crab, canned	122.0^f^	110–134	Muscles	37.7^c^	–	Short-necked clam	42.0^a^	32.7–49.7
Scallops (coral)	43.0^c^	–	Short-necked clam	15.1^a^	10.6–19.8	Octopus	31.7^a^	26.3–38.2
Cod, raw	28.0^b^	26.4–29.7	Sardine	11.7^d^	–	Muscles	26.3^c^	–
Scallops (white)	25.1^c^	–	Salmon, raw	6.5^d^	–	Tuna	16.6^a^	7.2–26.1
In tomato, canned	7.4	3.9–9.7	Trout	4^b^	3.9–4.2	Sardine	14.7^d^	–
Anchovies	5.0^g^	2.3–7.6	Salmon	3.7^a^	2.6–4.9	Horse mackerel	12.5^a^	6.6–19.6
Horse mackerel	4.7^a^	0.3–12.9	Octopus	3.6^a^	3.3–4.1	Scallops (coral)	10.1^c^	–
Shrimp	3.7^a^	–	Horse mackerel	3.0^a^	1.4–5.3	Squid	9.5^a^	5.1–14.9
Salmon, raw	3.3^d^	–	Mackerel, raw	2.9^f^	1.6–4.1	Salmon	9.1^a^	6.4–13.9
Salmon, raw	2.7^f^	1.6–4.6	Mackerel	2.7^a^	2.1–3.1	Trout	8.9^b^	8.7–9.1
Mackerel, raw	2.4^f^	1.3–3.5	Sardine	2.4^a^	2.1–2.9	Salmon, raw	8.2^d^	–
Short-necked clam	2.4^a^	1.7–2.9	Anchovies	2.2^g^	2.1–2.3	Anchovies	7.7^g^	7.5–7.9
Salmon	1.9	1.4–2.6	Scallops (coral)	2.1^c^	–	Sardine	5.6^a^	3.8–7.2
Trout	1.8^b^	1.8–1.9	Tuna	2.0^a^	0.5–3.1	Mackerel	4.2^a^	1.6–6.8
Cod, raw	1.4^b^	0.5–3.1	Salmon, raw	1.5^f^	0.4–3.3	Mackerel, raw	3.0^f^	0–7.7
Sardine	1.1^d^	–	Cod, raw	1.3^b^	1–1.6	Scallops (white)	2.8^c^	–
Octopus	1.1^a^	0.7–1.4	Crab, canned	1.4^f^	1.2–1.5	Crab, canned	2.1^f^	2-2.2
Muscles	1.0^c^	–	Scallops (white)	1.0^c^	–	Fish sausage	1.8^a^	1.0-2.6
Fish sausage	0.9^a^	0.3–1.2	Fish sausage	0.8^a^	0.2–1.0	Japanese needlefish	1.0^a^	0-3.0351
Mackerel	0.8^a^	0.4–1.2	Cod, raw	0.6^f^	0–3.8	Salmon, raw	0.8^f^	0-3.2
Sardine	0.8^a^	0.3–1.5	Cray fish	0.6^c^	–	Cod, raw	0.6^f^	0-2.2
Tuna	0.2^a^	0.1–0.2	Japanese needlefish	0.4^a^	0.1–0.8	Shrimp	0.4^a^	0.2–0.6
Cray fish	0.1^c^	–	Shrimp	0.1^a^	0.1–0.4			
Japanese needlefish	0.1^a^	0.1–0.2						
Squid	0.1^a^	0.1–0.2						
**CHEESE, MILK AND EGG**								
Cheddar, matured (1 year)^h^	653.0^b^	–	Cheddar, matured (1 year)^h^	199.5^b^	197–202	Cheese (processed)	1.4^a^	0.4–2.6
Blue, Norwegian	16.4^f^	12.6–20.2	Blue, Norwegian	23.8^f^	20.2–29.3	Yogurt, plain	0.8^f^	0–2.2
Blue, Japanese (1–5 months)	6.7^e^	–	Blue, Japanese (1–5 months)	20.3^e^	–	Blue, Norwegian	0.4^f^	0–2.0
Goat cheese (3 weeks)	0.6^c^	–	Cheese (processed)	4.3^a^	2.4–6.6	Boiled egg	0.4^b^	0.2–0.6
Yogurt, plain	0.3^f^	0	Camembert (3 weeks)^h^	1.5^e^	–	Full cream	0.4^b^	0.2–0.6
Boiled egg	0.3^b^	0.3–0.4	Yogurt, plain	0.7^f^	0	Semi-skimmed	0.3^b^	0.2–0.4
Chicken egg	0.3^a^	0–0.6	Goat cheese (3 weeks)^h^	0.6^c^	–	Goat cheese (3 weeks)^h^	0.3^c^	–
Semi-skimmed	0.2^b^	0.1–0.2	Semi-skimmed	0.5^b^	0.3–0.6	Chicken egg	0.2^a^	0–0.4
Soft cheese (4–8 weeks)	0.2^c^	–	Full cream	0.4^b^	0.2–0.5	Yogurt, plain	0.06^m^	–
Full cream	0.1^b^	–	Soft cheese (4–8 weeks)^h^	0.2^c^	–	Goat cheese (3 weeks)^h^		1.0–2.6
Yogurt, plain	0.1^m^	–	Boiled egg	0.2^b^	0–0.2	Chicken egg	0.2^a^	0–4.2
Milk (cow)	0.0^a^	0–0.1	Yogurt	0.15^a^	0–0.4			
Yogurt	0.0^a^	0–0.1	Chicken egg	0.15^a^	0–0.2			
			Yogurt, plain	0.07^m^	–			

a *Nishibori et al. (2007)*,

b *Bardócz et al. (1993)*,

c *Cipolla et al. (2007)*,

d *Nishimura et al. (2006)*,

e *Okamoto et al. (1997)*,

f *Eliassen et al. (2002)*,

g *Veciana-Nogues et al. (1997)*,

h *Dicáková et al. (2003)*,

i *Kozova et al. (2009a)*,

j *Silva and Gloria (2002)*,

k *Hernandez-Jover et al. (1997)*,

l *Yano et al. (1995)*,

m *Farriol et al. (2004). – Data not detected/data not available*.

### PAs in plant origin food

PAs in plant-based diet have been mostly studied in USA, Japan, UK, France, Germany, Spain, Sweden, and Norway. The levels of Put, Spd, and Spm in vegetables, fruits, grains and other plant-based foods are summarized in Table 1. Vegetables rich in Put include green pepper, dried soybean, green peas, eggplant and green onion. High amounts of Put, in the range of 31.2–84 mg kg^−1^ FW with a mean value of 54.7 mg kg^−1^ FW, were found in Japanese fresh green pepper (Nishibori et al., 2007). Spd- and Spm-rich vegetables are mushrooms, broccoli, lettuce and pumpkin, along with beans. Dried soybean (Put 41, Spd 207, and Spm 69.0, mg kg^−1^ FW) and green peas (Put 32.4, Spd 49.5, and Spm 6.5, mg kg^−1^ DW) are among the top 10 vegetables rich in all the three PAs (Okamoto et al., 1997; Nishibori et al., 2007). Overall, beans seem to have higher levels of Spd and Spm while many vegetables are higher in Put and Spd (Table 1). Orange, mango, and banana feature among the top ten fruits highly-enriched in PAs, with Put being the main PA followed by Spd (Bardócz et al., 1993; Okamoto et al., 1997; Eliassen et al., 2002; Nishimura et al., 2006; Table 1). Fruits other than these, such as lime (41.0 mg kg^−1^ FW Put), pear (24.0 mg kg^−1^ FW Put) and melon (11.7 mg kg^−1^ FW Spd) are also good sources of PAs (Bardócz et al., 1993; Nishimura et al., 2006).

Cereals are among the most staple source of carbohydrates and fiber for human diet. Notably, maize (corn), brown rice and whole grain wheat are richer in Spd (Bardócz et al., 1993; Okamoto et al., 1997; Eliassen et al., 2002; Nishimura et al., 2006; Nishibori et al., 2007), with the following decreasing order: Japanese corn (43.0 mg kg^−1^ FW) > whole grain wheat (13.1–21.0 mg kg^−1^ FW) > millet (9.1 mg kg^−1^ FW) > brown rice (6.4 mg/kg ^−1^FW). Interestingly, all these grains contain more Spm than Put. In addition to vegetables, fruits and grains, nuts are also a good source of PAs. Pistachio and almonds contain high levels of all three major PAs (Nishibori et al., 2007) whereas hazelnut (Cipolla et al., 2007) and cashew (Nishibori et al., 2007) contain higher amounts of Spd (21 mg kg^−1^ FW) and Spm (24 mg kg^−1^ FW), respectively.

In the context of common beverages, tea and coffee are the two most favorite beverages consumed by humans. Black tea leaves are rich in Spm (59 mg kg^−1^) followed by Spd (38.1 mg kg^−1^) and Put (15.3 mg kg^−1^) while the pattern of PAs content in green coffee was found to be in the reverse order, with Put at 10.3 mg kg^−1^ significantly higher than Spd (6.0 mg kg^−1^) and Spm (4.4 mg kg^−1^), respectively (Bardócz et al., 1993; Cirilo et al., 2003).

In certain diets, spices are an integral part where they not only provide flavor but also are thought to help fight infection, boost immune system, reduce inflammation, and prevent cancer and heart diseases. Among such spices, peppermint contains 2 mg kg^−1^ Spm and dill contains 29.2 mg kg^−1^ Spd (Nishimura et al., 2006).

### PAs in animal origin food

PA content in meats, seafood and dairy products is summarized in Table 2. Many studies have indicated that Put levels in animal meat are generally lower (less than 10 mg kg^−1^) as compared to Spm (more than 25 mg kg^−1^) (Bardócz et al., 1993; Yano et al., 1995; Hernandez-Jover et al., 1997; Okamoto et al., 1997; Veciana-Nogues et al., 1997; Eliassen et al., 2002; Silva and Gloria, 2002; Dicáková et al., 2003; Farriol et al., 2004; Nishimura et al., 2006; Cipolla et al., 2007; Nishibori et al., 2007; Kozova et al., 2009b). Pheasant, duck, deer, chicken, lamb and pork contain higher levels of Spm compared to Spd and Put, with beef liver containing the highest levels, 197 mg kg^−1^ FW, of Spm (Nishimura et al., 2006). However, fallow deer contains higher levels of Put (38 mg kg^−1^ FW) than Spd (14.7 mg kg^−1^ FW) (Dicáková et al., 2003). Among the seafood—canned crab, coral scallops, raw cod, and shrimp do not follow any typical pattern, i.e., high Spm and low Put, except they are richer in Put (Bardócz et al., 1993; Eliassen et al., 2002; Cipolla et al., 2007). On the other hand, short-necked clam, muscles, octopus, salmon and sardine are rich sources of Spm and Spd while canned crab (122 mg.kg^−1^), scallops (43 mg.kg^−1^), raw cod (28 mg.kg^−1^), and shrimp (3.7 mg.kg^−1^) are more enriched in Put (Ali et al., 2011). Spm levels in meat and meat products of warm-blooded animals are generally higher, ranging from 20 and 60 mg kg^−1^ whereas fish contain < 10 mg kg^−1^ (Kalac and Krausova, 2005). It is apparent that high concentrations of Spd and Spm is typical of foods from animal origin as compared to the plant products (Kalac and Krausova, 2005).

Also, extremely high levels of Put and Spm are present in matured cheddar cheese (Dicáková et al., 2003), Blue Norwegian cheese (Eliassen et al., 2002) and Blue Japanese cheese (Okamoto et al., 1997). In comparison, PA concentration is low in milk, yogurt, and eggs (Bardócz et al., 1993; Farriol et al., 2004; Nishimura et al., 2006).

## Storage and processing of tissues/cells affect PA content

Storage of leafy vegetables—Chinese cabbage, endive, iceberg lettuce and radicchio, at 5°C for more than 5 days increased their Put levels from 3- to 8-fold without any significant change in Spd and Spm levels (Simon-Sarkadi et al., 1994). Significant reduction in the levels of Spd with a complete loss of Put and Spm was achieved after green coffee was roasted (Cirilo et al., 2003). On the other hand, the fermentation of soy-based products results in elevated levels of PAs (Okamoto et al., 1997). Irradiation of Korean soybean paste before fermentation decreased the levels of Put and Spd but not Spm (Kim et al., 2003). Similarly, cooking of vegetables—broccoli, cauliflower, savoy, and asparagus causes significant losses in PA levels due to leaching in water (Ziegler et al., 1994).

A very elaborate information about free and conjugated PAs in fermented foods consumed by Japanese is also available (Okamoto et al., 1997). In short, the typical fermented food items, miso and soy sauce, were found to be high in Put and Spd but almost deficient in Spm. The differences between PAs profiles of soy sauces and soybeans confirmed that PAs are produced upon fermentation, due to microbial decarboxylation. In comparison to soy sauce and miso, natto and tempe—relatively simple forms of fermented soybean—possess lesser amounts of PAs than soybean due to the degradation of some PAs during fermentation.

PAs in beef, pork and lamb stored at 5°C have also been analyzed (Edwards et al., 1983). Put levels increased over 13 days of storage. However, when vacuum-packed beef sirloin was stored up to 39 days at 0, 5, or 10°C, Spd and Spm contents did not change (Yano et al., 1995). In summary, storage and processing—be it fermentation, roasting, ripening or cooking, results in alterations in the levels and accumulation patterns of specific PAs.

## Homeostatic regulation of PAs and H_2_O_2_ evolution in plants

Cellular PA homeostasis is achieved via interconversion of PAs, process catalyzed by PA amine oxidase (PAO) and diamine oxidase (DAO) enzymes. Such enzymatic reactions lead to PA catabolism and also the production of H_2_O_2_ as one of the products (Figure 1). PAO/DAO catalysis that generates H_2_O_2_ from PAs adds another dimension to the function of PAs in living cells, particularly because of the potential of H_2_O_2_ both as a secondary messenger and a signaling molecule (Cona et al., 2006; Tavladoraki et al., 2012). In the back-conversion reactions, Spm is oxidized to Spd, and Spd can also be oxidized to Put (Figure 1).

PAOs catalyze flavine adenine nucleotide (FAD)-dependent oxidation of Spd and Spm and/or their acetylated forms while the preferred substrate for copper amine oxidases (CuAOs) is Put (Cona et al., 2006; Tavladoraki et al., 2012). Five PAO genes (AtPAOs) and ten CuAO genes (AtCuAOs) are present in a model plant Arabidopsis (Fincato et al., 2011; Planas-Portell et al., 2013; Ahou et al., 2014; Kim et al., 2014). The functions of PAOs include light-induced inhibition of the mesocotyl growth (Cona et al., 2003), cell death (Tisi et al., 2011a), wound-healing (Angelini et al., 2008), salt stress (Moschou et al., 2008), JA-induced root xylem differentiation (Ghuge et al., 2015), and pathogen attack (Moschou et al., 2009) to name a few. PA-derived H_2_O_2_ triggers signal transduction pathways causing defense gene expression, stress tolerance, or cell death (Moschou et al., 2008; Tisi et al., 2011b). ROS signaling involving oxidative PA catabolism is situational, for instance, during stress where plant growth is under stress (Ghuge et al., 2015). The apoplastic copper amine oxidase AtAO1 mediates jasmonic acid-induced protoxylem differentiation in Arabidopsis roots. Another metabolic salvage pathway associated with DAO-mediated oxidation of Put which generates pyrroline which is metabolized to γ-aminobutyric acid (GABA) and the latter feeds into Krebs cycle and N metabolism (Figure 1).

## PAs and abiotic stress in plants

Progress in elucidating role(s) of PAs in plants was catalyzed when gain-of-function and loss-of-function genetic plants with modified levels and/or the type of PA were developed and analyzed (Noh and Minocha, 1994; Kumar et al., 1996; Mehta et al., 2002; Capell et al., 2004; Nambeesan et al., 2010, 2012; Majumdar et al., 2013). These transgenic approaches brought to light PA roles in plants as follows: extending fruit life *on planta*, delayed seasonal whole plant senescence, enhanced anabolic and N-C cellular interactions Mehta et al., 2002; Mattoo et al., 2006; Nambeesan et al., 2010), accumulation of anti-cancer molecules such as lycopene (Mehta et al., 2002), role of Orn in regulating PA homeostasis and Glu levels (Majumdar et al., 2013); and tolerance to abiotic stresses including drought and salinity (Capell et al., 2004; Cona et al., 2006; Kusano et al., 2007; Alcazar et al., 2010b; Shukla and Mattoo, 2013; Minocha et al., 2014; Mattoo et al., 2015; Pal et al., 2015). Thus, transgenic tobacco, rice, and tomato plants engineered to accumulate PAs by overexpression of SAM decarboxylase showed tolerance against salt, osmotic, and heat stresses (Wi et al., 2006; Alcazar et al., 2010a), and engineered Spd overexpression in Arabidopsis, pear and potato plants enabled these transgenic plants to be tolerant against drought, salt and oxidative stresses (Kasukabe et al., 2004, 2006; He et al., 2008; Wen et al., 2008; Alcazar et al., 2010a). In contrast, and as a confirmation of the above findings, PA pathway-deficient Arabidopsis mutants were more sensitive to salt stress (Watson et al., 1998; Kasinathan and Wingler, 2004; Urano et al., 2004; Yamaguchi et al., 2006).

## PAs and human health

### Phenotypes associated with polyamine-deficient mutants

Functions of PAs in mammals has been recently reviewed (Pegg, 2016). Since polyamines play significant roles in various physiological processes, mutations resulting in the loss of either Put, Spd, or Spm have been linked to various phenotypes in many organisms including humans. Spd is the precursor of hypusine which is required for the post-translational modification of the elongation factor eIF5A and its deficiency thereof affects protein function (Pegg, 2016). The function of Spm emerged with the characterization of a male offspring of a mice mutant, designated as Gyro, gene sumbol Gy (Lyon et al., 1986). This mutant was deficient in the synthesis of Spm, due to a deletion in the Spm synthase (SpmSyn) gene along with a part of the adjacent *Phex* gene. This mutation led to several phenotypes, including hypophosphatemia with rickets/osteomalacia and circling behavior, reduced size, sterility, deafness, neurological abnormalities, and short life span due to sudden death (Lyon et al., 1986; Lorenz et al., 1998). The hypophosphatemia and reduced size in Gy mice were associated with the partial loss of the Phex gene, while other phenotypes were attributed to the reduced levels of Spm (Meyer et al., 1998). Essential role of Spm in humans became apparent after molecular characterization of an X-linked recessive condition in males associated with intellectual disability disorder known as Snyder-Robinson syndrome (SRS) (Albert et al., 2013). The SRS is also associated with osteoporosis, dysmorphic faces, speech and gait abnormalities, seizures, muscle hypoplasia, and kyphoscoliosis (Albert et al., 2013). The SRS syndrome was shown to result from the inaccurate splicing of SpmSyn mRNA, causing production of inactive truncated protein and low levels Spm (Cason et al., 2003).

### Aging

Cellular levels of Spd and Spm decrease during aging of many organisms (Nishimura et al., 2006; Minois et al., 2011; Zwighaft et al., 2015). This decline in PA levels plays significant role in the aging process of organisms, from bacteria, fungi, plant, to mammals (Eisenberg et al., 2009; Nambeesan et al., 2010). Spd levels in human population are variable, it is higher in those under 50 years of age, lower in those within 60–80 years of age, while those who were 90 years or older had Spd levels similar to the younger population (<50 years). These results were interpreted to suggest that higher Spd levels play a role in human longevity (Pucciarelli et al., 2012; Minois, 2014). Studies using dietary supplementation with exogenous Spd, which ameliorated many of the deleterious physiological effects of aging in mice, fruit fly and *C. elegance* and led to 15–40% increase in their life span, have supported the contention that Spd is good for healthy subjects (Eisenberg et al., 2009; Soda et al., 2009a,b). Similarly, addition of Spd to aging yeast cultures resulted in about 4-times increase in their clonogenic survival while similar treatment of human peripheral blood mononuclear cells increased their viability from 15 to 50% in a 12-days old culture cells (Eisenberg et al., 2009). Also, incidence of age-related kidney glomerular atrophy and DNA methylation were found to decrease with high PA diet fed to mice (Soda et al., 2009a,b). Exogenous Spd also reduced the age-associated overproduction of reactive oxygen and oxidative damage in yeast, fruit fly and mice, and imparted heat tolerance to yeast and oxidative stress tolerance to fruit fly (Eisenberg et al., 2009; Soda et al., 2009a,b). In plants, engineering high endogenous Spd and Spm levels prolonged the phase of tomato fruit ripening, the terminal developmental stage in the ontogeny of fruit, and increased fruit shelf life up to 40% compared to low-PA fruit (Mehta et al., 2002; Nambeesan et al., 2010). Enhanced autophagy by higher PA levels has been suggested to be the primary mechanism by which PAs affect the aging process (Eisenberg et al., 2009).

Many organisms, including *Drosophila*, mouse and human, show age-dependent impairment in memory that is associated with a decrease in PAs, especially Put and Spd. Although the molecular basis of such impairment is not known, PAs have been shown to play essential role in both aging and age-associated memory loss. Spd feeding to aging fruit flies restored juvenile PA levels concomitant with recovery of the age-induced memory (Gupta et al., 2013). In addition to the restoration of memory loss, the Spd-fed fruit flies were found to undergo higher levels of autophagy. Both of these processes remained impaired in the fruit flies that carried a mutation in the autophagy process. The ectopic expression of ODC gene under neuron-specific appl-Gal4 promoter protected fruit flies against age-induced memory loss. Taken together, these results indicate a role of Spd-induced autophagy in resorting the age- associated memory loss (Gupta et al., 2013). In addition, these results provide evidence for the diet-based PA acquisition for maintaining memory during aging.

Effects of higher PAs in general and Spd in particular on aging have been attributed to several biochemical mechanisms including increased autophagy, lipid metabolism, cell growth and death processes. Autophagy plays a positive role in slowing the aging process by removing the damaged proteins and organelles from cells (Yamaguchi and Otsu, 2012). PAs, particularly Spd, were shown to increase autophagy in many organisms, implicating them in enhancing their life-span (Eisenberg et al., 2009; Minois et al., 2014). Spd was also found to reduce necrosis in yeast by inhibiting aging, one of the factors considered to regulate this process (Eisenberg et al., 2009). Spd inhibits histone acetylases, thereby increasing longevity by enhancing cytoprotection, a mechanism similar to that suggested for an antioxidant resveratrol that activates histone deacetylase Sirtuin 1 to confer longevity (Morselli et al., 2010). However, it remains to be determined if the same histones are targets for both Spd and resveratrol.

### Memory

#### Preexisting memory

Seminal neurochemical and neuro-physiological studies have provided a wealth of information on the role of PAs in the central nervous system (Seiler and Schmidt-Glenewinkel, 1975; Ransom and Stec, 1988; Sacaan and Johnson, 1990; Williams et al., 1994; Shaw et al., 2001). The *N*-methyl-D-aspartate (NMDA)-glutamate receptor plays a role in spatial learning which became apparent when infusion of its antagonists selectively impaired learning (Morris et al., 1986). Dizocilpine (MK-801) is an uncompetitive antagonist of NMDAr and is reported to impair learning. By interacting with NMDAr, Spd potentiates the effect of MK-801 on learning impairment (Shimada et al., 1995). Spd also attenuated working memory acquisition deficit induced by intrahippocampal MK-801, scopolamine (muscarinic antagonist) and AIDA (a metabotropic glutamate receptor class I antagonist) suggesting that Spd affects memory by binding to NMDAr (Kishi et al., 1998a,b). Earlier it had been shown that brain Spd, but not Spm, levels increased during the social-isolation associated aggressive behavior and decreased upon transferring the animal to its colony (Tadano et al., 2004). Other studies have reported that higher levels of PAs, specifically Spm, were toxic to sedation, hypothermia, anorexia, adipsia, seizures and focal encephalomalacic lesions (Rosenthal et al., 1952; Anderson et al., 1975). An intracerebroventricular treatment with 125 nmol Spm potentiated the deleterious effects of diazepam and abolished previously-learned platform position (Conway, 1998).

#### Trained learning and memory

Many studies have focused on the effects of Spd on shock-motivated learning and memory including during stepdown inhibitory and passive avoidance tasks, fear conditioning acquisition and consolidation. Intraperitoneal, intracerebroventricular, intrahippocampal, or intra-amygdala delivery of Spd (0.02–20 nmol) improved early consolidation during stepdown and inhibitory avoidance tasks (Guerra and Rubin, 2016). Arcaine (a potent antagonist for the PA binding site on the NMDAr) at 0.002–0.2 nmol had either no effect or prevented Spd-associated consolidation during stepdown and inhibitory avoidance tasks. Spd (0.02–2 nmol) generally improved fear conditioning acquisition and consolidation whereas arcaine (0.0002–0.02 nmol or 10–30 mg/kg) treatment impaired this acquisition (Rubin et al., 2004; Camera et al., 2007; Da Rosa et al., 2012; Signor et al., 2014; Guerra and Rubin, 2016 and references therein). PAs also modify reconsolidation and extinction of fear conditioning and step-down avoidance, but the observed effects evaluated by different investigators were variable. The intraahippocampal treatment with 2 nmol Spd facilitated fear extinction in one investigation (Gomes et al., 2010) but not in another investigation at 10 nmol Spd (Bonini et al., 2011). Yet in another investigation, the intraahippocampal Spd treatment at 0.02–0.2 nmol improved memory consolidation. PAs generally potentiated the deleterious effects of diazepam MK-801 as determined by Morris-water maze, object recognition and place conditioning. However, the intraperitoneal injection of 5.0–20 mg/kg Spd improved social memory. Spm at 10–125 nmol potentiated detrimental effects of diazepam and tended to impair objective recognition memory (see Guerra and Rubin, 2016 and references herein).

### Cancer

PAs are well known to be involved in cell division, proliferation, and pro-growth metabolism that can cause proliferation in normal cells (Slocum and Flores, 1991; Cohen, 1998; Cassol and Mattoo, 2003; Kaur-Sawhney et al., 2003; Janne et al., 2004). Although there is no direct evidence that PAs as such can initiate cancer, caution has been expressed to their deployment/ingestion in situations where the cells suffer from uncontrolled growth and disease, for example cancer. Interest in PAs as promoters of cellular proliferation and growth developed after reports on the presence of ODC activity in regenerating rat liver, chick embryo, and various tumors (Russell and Snyder, 1968) and increase in PAs levels in patients with familial adenomatous polyposis (FAP), a genetic form of colon cancer (Giardiello et al., 1997). The observation that cancerous cells and liver of tumor-bearing mice (Andersson and Heby, 1972), and cancerous colon, breast and skin (Upp et al., 1988; Manni et al., 1995; Gilmour, 2007) accumulate elevated levels of PAs suggested their possible association with cancerous cells (Wallace and Caslake, 2001). These studies added further caution about feeding PAs or diets rich in them to patients with growth abnormalities. This led to a rationale put forth to use an inhibitor of ODC, difluoromethylornithine (DFMO), as a chemopreventive agent (Nowotarski et al., 2013). Thus, reduction of exogenous PAs from food and gut microbiome, or inhibition of PAs biosynthesis, are some strategies in vogue to test their usefulness during chemotherapy.

Several studies have been conducted where tumor-bearing animals were fed a Polyamine Reduced/Deficient Diet (PRD/PDD) or treated with DFMO and neomycin (for partial decontamination of the gastro-intestinal tract) to determine the effects of these treatments on remission/ reduction of tumor in these animals. The results from these investigations showed significant inhibition of tumor progression and spreading metastasis, as well as stimulation in anticancer immunity, without inducing deleterious secondary effects (Seiler et al., 1990; Chamaillard et al., 1997). The tumor-grafted animals fed only on PDD together with neomycin in the drinking water (i.e., without DFMO) also exhibited about 40% inhibition in tumor progression and metastasis spreading (Seiler et al., 1990). Neither neomycin nor DFMO was able to positively modify the malignant evolution. Thus, such positive data led to clinical trials with a PA-free oral nutritional supplement (ONS) combined with docetaxel fed to castrate-resistant prostate cancer (CRPC) patients, but yielded little side effects (Artignan et al., 2012). It has been suggested that PAs do not trigger cancer, but accelerate tumor growth (Kalač, 2014).

#### Innate immunity

Several factors modulate innate immunity in animal models (Brubaker et al., 2015). These include structural components of bacteria, fungi, virus, and their metabolites, such as nucleic acids that activate germline-encoded pattern-recognition receptors (PRRs) to induce/enhance innate immunity (Medzhitov, 2009). Most PRRs are divided into 6 families designated as Toll-like receptors (TLRs), C-type lectin receptors (CLRs), nucleotide binding domain, leucine-rich repeat (LRR)-containing (or NOD-like) receptors (NLRs), RIG-like receptors (RLRs), and AIM2-like receptors (ALRs). These families are further subdivided into membrane bound and free intracellular receptors (Brubaker et al., 2015). The PRRs act primarily as transcriptional regulators for the production of cytokines and interferons (IFN) - the proinflammatory chemical messages that initiate innate and adaptive immune responses. Some PRRs also initiate nontranscriptional responses, including phagocytosis, autophagy, cell death, and cytokine processing (Deretic et al., 2013; Lamkanfi and Dixit, 2014). Signal transduction pathways tightly control the transcriptional as well as nontranscriptional innate immune responses by PRR-mediated microbial detection (Palm and Medzhitov, 2009).

PAs modify the innate immune responses by activating TLRs that detect various microbes in the vicinity and activate innate immunity after infection (Medzhitov, 2001). The TLRs represent a family of single membrane-spanning non-catalytic receptor proteins capable of recognizing structurally conserved microbial molecules such as lipopolysaccharides (LPS) (Pirnes-Karhu et al., 2012). Interestingly, LPS are reported to induce expression of ODC in neurons and microglia across the mouse central nervous system (CNS) and increase transiently the transcription of genes encoding pro-inflammatory cytokines and TLR2 in microglial cells (Soulet and Rivest, 2003). Inhibition of ODC activity by its inhibitor DMFO ameliorated the LPS-associated increase in ODC and lowered Put levels in cells, resulting in increased survival rate of mice by abolishing neurodegeneration after treatment with the endotoxin. These findings suggest that PAs play a role in maintaining the neuronal integrity and cerebral homeostasis during immune insults (Soulet and Rivest, 2003). Although both TLR2 and TLR4 are implicated in the innate immune response of the intestinal epithelial cells to bacterial pathogens, only an increase in TLR2 transcripts with concomitant increase in epithelial barrier function was observed upon the ectopic expression of ODC and this increase was greatly reduced by DMFO treatment, an ODC inhibitor (Chen et al., 2007). Significant changes in the levels of TRL4 transcripts were not obtained by either decreasing or increasing the function of ODC suggesting that PAs specifically activate TLR2 expression that regulates the epithelial barrier function (Chen et al., 2007). Collectively, these results provide support to the hypothesis that PAs play a role in LPS-induced innate immunity, although more research is needed to establish the role(s) for various PAs present in the cellular milieu. The generation of ROS has been implicated in the activation of innate immunity of gastric epithelial cells and macrophages to counteract infection by *Helicobacter pylori* since H_2_O_2_ produced during the back conversion of Spd and Spm enhanced protection to gastric epithelial cells and macrophages against *H. pylori* (Gobert and Wilson, 2017). However, the ROS-mediated oxidative DNA damage and resulting mutations might also play a role in colonizing *H. pylori* to gastric niche (Gobert and Wilson, 2017). T-cells in aged mice exhibit decreased autophagy in the lysosomal degradation pathway leading to their decay. However, an antibacterial molecule from the hemocytes of the spider *Acanthoscurria gomesiana* has been reported to enhance the innate immune response in splenocytes by inducing IFN-γ and NO synthesis (Mafra et al., 2012).

#### Cognitive impairments

PA effects on cognitive memory, including quinolinic acid (QA)-induced, lipopolysaccharide (LPS)-induced, amyloid peptide (A)-induced, and brain trauma-induced cognitive deficit in animal model systems have been reviewed recently (Guerra and Rubin, 2016). These studies have begun to provide further evidence that endogenous or dietary PAs can have either beneficial or deleterious effects on cognitive diseases depending upon physiological or pathological impairment of memory (Frühauf et al., 2015). Therefore, it appears that diets rich in PAs should have beneficial effects on “early” inflammation and memory impairment but may be harmful once the pathological conditions have set in.

#### Quinolinic acid (QA)-induced memory impairment

Huntington's disease (HD), a fatal genetic disorder, is associated with progressive breakdown of nerve cells in the brain causing memory deficits. Intrastriatal injection with quinolinic acid (QA) has provided animal model for this disease because it duplicates many histopathological and neurochemical symptoms and neurofunction loss of HD (DiFiglia, 1990). Intrastriatal injection with a 10 nmol Spm dose impaired object recognition after training in rodents, whereas a low dose of Spm (0.1 nmol) reverted the deleterious effect of a QA injection, attenuating QA-induced astrogliosis (Velloso et al., 2009). These results were interpreted to indicate that low Spm dose increases NMDAr activity in the striatum but an overdose decreases them (Velloso et al., 2009). Brain trauma-induced cognitive deficit increase in newly born astrocytes was reversed by feeding DFMO in the drinking water (Rosi et al., 2012). Interestingly, astrogliosis decreased during the reversal of QA-associated memory impairment (Velloso et al., 2009).

Lipopolysaccharide-induced neuroinflammation associated memory impairment LPS's are cell-wall components of gram-negative bacteria and induce neuroinflammation in the cerebral cortex and hippocampus. LPS-induced neuroinflammation that results in memory impairment is associated with spatial memory, contextual fear conditioning, and avoidance learning in rats and mice (Shaw et al., 2001; Lee et al., 2012). This type of neuroinflammation was found to be associated with increases in cytokines such as IFN-γ, IL-6, IL1-β, and TNFα levels. The intraperitoneally injected Spd (0.3 mg/kg) reversed the LPS-induced inflammation and memory impairment but not the LPS-induced increase in cytokines (Frühauf et al., 2015). Once again, involvement of Spd regulation of NMDAr activation was the reason given for these observed results (Li and Tsien, 2009). Glutamate and glycine/D-serine-mediated activation of NMDAr increases the flow of the positively charged ions through the cell membrane and, in the process, affects memory (Furukawa et al., 2005). However, the expression of these receptors is sensitive to the neuro-inflammatory challenges, affecting long-term NMDA-dependent potentiation in the hippocampus in the LPS-treated tissue (Min et al., 2009). Put, Spd, and Spm bind to the lower lobe of the N-terminal domain of GluN1 and GluN2B dimer interface, and allosterically activate NMDAr and impairing memory (Mony et al., 2011). Tenprodil, an inhibitor of the NMDAr, eliminates the protective effect of Spm, supporting this hypothesis. Collectively, these results, provide further evidence that NMDAr mediate the LPS-induced cognitive impairment (Frühauf et al., 2015).

## Alzheimer and parkinson diseases

Alzheimer (AD) and Parkinson (PD) diseases are generally associated with aging and cause progressive decline of cognitive function. These severe neurological impairments are associated with accumulation of phosphorylated *tau* protein that forms neurofibrillary tangles and neurotoxic amyloid beta-peptide (Aβ), which is responsible for the formation of the senile plaques (Roberson and Mucke, 2006). The AD patients exhibit increase in the activity of ODC and PA levels in the brain, which had been implicated in the PAs role in both cognitive deficit and synaptic loss (Morrison et al., 1998; Inoue et al., 2013). Exposure of neuronal cell cultures to Aβ increased PA levels, NMDAr activation, and synaptic loss (Yatin et al., 2001). The intracerebral injection of Aβ induced cognitive impairment in experimental animals, which was reversed by NMDA antagonists, suggested a role in memory loss (Klyubin et al., 2011). Also, blocking the PA binding site in NDMAr either by arcaine or inhibiting PA synthesis by DFMO reversed the Aβ25-35-induced memory impairment in mice (Gross et al., 2013). Collectively, these studies provide evidence that PAs are deleterious to Aβ-accumulation and higher PA levels under these conditions cause cognitive decline (Inoue et al., 2013), a stipulation opposite to the effects of high PAs in improving learning and memory in naive animals. Parkinson's disease reduces expression of SAT1, a catabolic PA enzyme, and consequently higher PAs levels increased in patients. This increase in PAs levels has been implicated in cognitive reduction in Parkinson patients via NMDAr pathway (Lewandowski et al., 2010). Higher PA levels in Parkinson patients are also associated with the aggregation of α-synuclein, but its role in Parkinson's disease is not yet known.

## Conclusions

**Figure 2:**
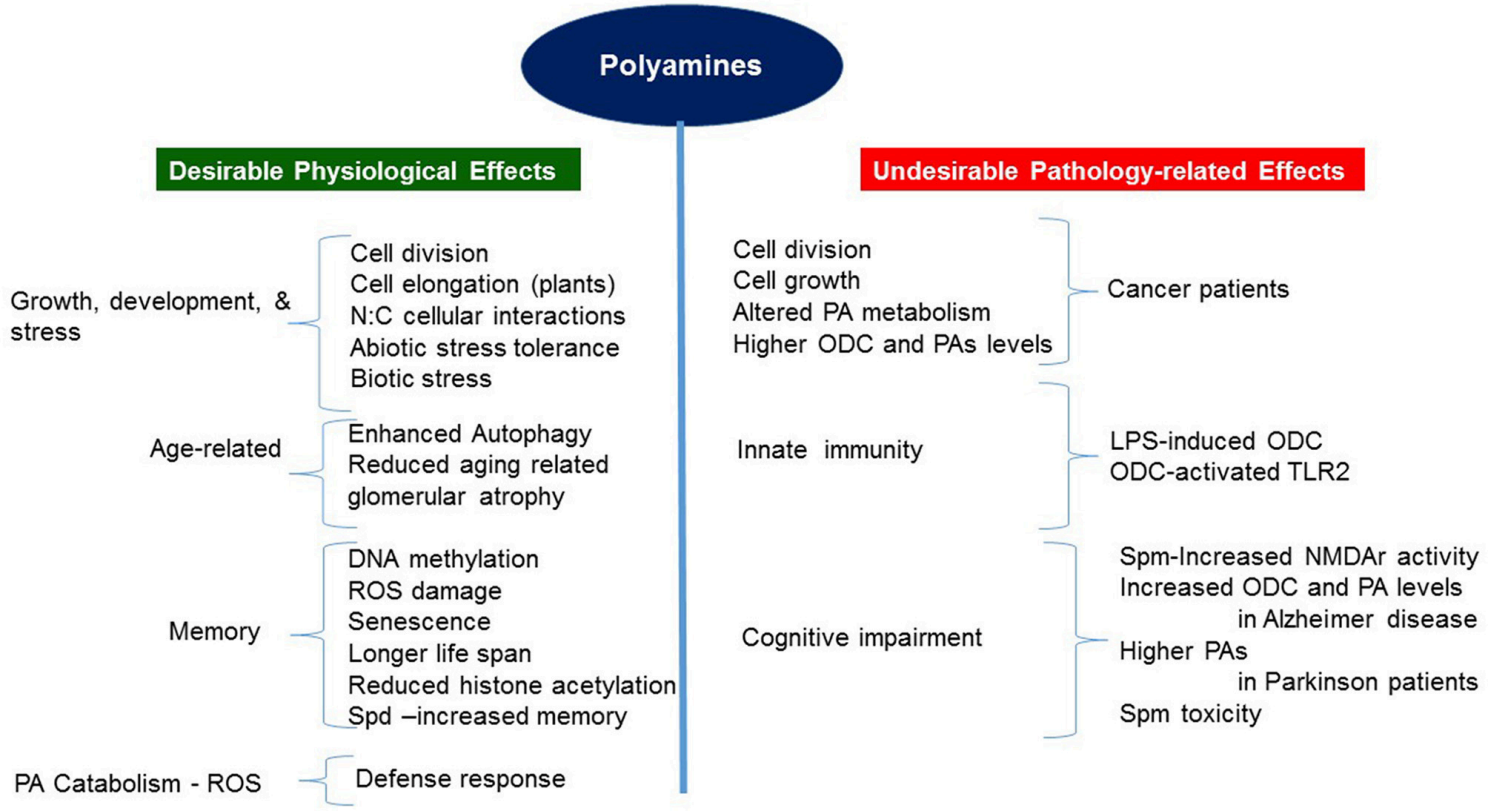
Positive and negative aspects of polyamine function/role in plants and humans/animals.

PAs play essential roles in a wide range of biochemical and physiological processes during growth and development of both mammals and plants, as also in their cellular responses to biotic and abiotic stresses (summarized in Figure 2). Our current understanding of the roles PAs play was intensified and emerged through genetic evidence obtained through the characterization of mutations in the PA biosynthetic pathway and analyzing molecular, biochemical and physiological changes in specific transgenic organisms altered in the levels of a particular PA or PAs (Mehta et al., 2002; Mattoo et al., 2006, 2010; Sriva et al., 2007; Sobolev et al., 2014; Kusano and Suzuki, 2015; Fatima et al., 2016; Goyal et al., 2016; Pegg, 2016). It has become apparent that PA function is dependent upon the cellular level of each PA, likely differentiable and specific to each diamine, triamine and tetramine (Put, Spd, and Spm, respectively) (Handa and Mattoo, 2010; Mattoo et al., 2010), and in many instances being temperature-dependent (Mattoo, 2014; Liu et al., 2015; Goyal et al., 2016). Further understanding of how living cells achieve PA homeostasis and which master regulators regulate their biosynthesis, interconversion, catabolism and conjugation to bring about desirable biologically active cellular concentrations to impact specific biological processes in normal and stressful environment(s) is necessary to fully appreciate their functions in different biological systems. In plants, for the most part, PAs seem to play positive roles in normal cellular functions, for instance, in increasing longevity, increasing pro-health carotenoids such as lycopene, recalling physiological memory, enhancing carbon and nitrogen resource allocation/signaling, as well as in plant development and responses to extreme environment.

Thus far, research has established that PAs generally boost physiological processes to reduce aging, stress-induced responses and loss of memory, but detrimentally influence pathology-related conditions such as cancer, Alzheimer and Parkinson diseases (Pegg, 2016). Genetic evidence for the positive PA roles in mammals is associated with a number of growth and developmental processes, including the behavioral aspects, and emerged through studies that characterized mice and human mutations with defective production of Spm (Lyon et al., 1986; Cason et al., 2003; Albert et al., 2013). Mechanistically, some functions of PAs are conserved, providing parallels between plants and humans. One such conserved parallel is the connection between PAs and the signaling TOR function in plants and humans (Ren et al., 2012; Zabala-Letona et al., 2017).

A beneficial role of dietary Spd in enhancing life span of various organisms and human cell lines tightens the connection between “wellness diets” and human health (Eisenberg et al., 2009). Emerging understanding of PA action should open up new vistas not only for human health but also the plant life. Interestingly, oral Spd-supplemented diet given to mice extended their life span and exerted cardioprotective effects, including reduced cardiac hypertrophy and preserving diastolic function in old mice (Eisenberg et al., 2016). A research survey based on dietary intake of Spd levels suggested a correlation of Spd levels with reduced blood pressure and a lower incidence of cardiovascular disease (Eisenberg et al., 2016). These positive features of higher PA Spd in enhancing quality of life both in animals/humans and plants alike augment well for the future development of PA-based therapies.

## Author contributions

AH: outlined the review; AH, TF, and AM: each wrote sections; AM: finalized the review.

### Conflict of interest statement

The authors declare that the research was conducted in the absence of any commercial or financial relationships that could be construed as a potential conflict of interest.
